# Antiaging, Stress Resistance, and Neuroprotective Efficacies of *Cleistocalyx nervosum* var. *paniala* Fruit Extracts Using *Caenorhabditis elegans* Model

**DOI:** 10.1155/2019/7024785

**Published:** 2019-11-29

**Authors:** Mani Iyer Prasanth, James Michael Brimson, Siriporn Chuchawankul, Monruedee Sukprasansap, Tewin Tencomnao

**Affiliations:** ^1^Age-Related Inflammation and Degeneration Research Unit, Department of Clinical Chemistry, Faculty of Allied Health Sciences, Chulalongkorn University, Bangkok 10330, Thailand; ^2^Immunomodulation of Natural Products Research Group, Faculty of Allied Health Sciences, Chulalongkorn University, Bangkok 10330, Thailand; ^3^Department of Transfusion Medicine and Clinical Microbiology, Faculty of Allied Health Sciences, Chulalongkorn University, Bangkok 10330, Thailand; ^4^Food Toxicology Unit, Institute of Nutrition, Mahidol University, Salaya, Putthamonthon, Nakhon Pathom 73170, Thailand

## Abstract

Plant parts and their bioactive compounds are widely used by mankind for their health benefits. *Cleistocalyx nervosum* var. *paniala* is one berry fruit, native to Thailand, known to exhibit various health benefits *in vitro*. The present study was focused on analyzing the antiaging, stress resistance, and neuroprotective effects of *C. nervosum* in model system *Caenorhabditis elegans* using physiological assays, fluorescent imaging, and qPCR analysis. The results suggest that the fruit extract was able to significantly extend the median and maximum lifespan of the nematode. It could also extend the healthspan by reducing the accumulation of the “age pigment” lipofuscin, inside the nematode along with regulating the expression of *col-19*, *egl-8*, *egl-30*, *dgk-1*, and *goa-1* genes. Further, the extracts upregulated the expression of *daf-16* while downregulating the expression of *daf-2* and *age-1* in wild-type nematodes. Interestingly, it could extend the lifespan in DAF-16 mutants suggesting that the extension of lifespan and healthspan was dependent and independent of DAF-16-mediated pathway. The fruit extract was also observed to reduce the level of Reactive Oxygen Species (ROS) inside the nematode during oxidative stress. The qPCR analysis suggests the involvement of *skn-1* and *sir-2.1* in initiating stress resistance by activating the antioxidant mechanism. Additionally, the fruit could also elicit neuroprotection as it could extend the median and maximum lifespan of transgenic strain integrated with A*β*. SKN-1 could play a pivotal role in establishing the antiaging, stress resistance, and neuroprotective effect of *C. nervosum.* Overall, *C. nervosum* can be used as a nutraceutical in the food industry which could offer potential health benefits.

## 1. Introduction

Plants can synthesize many bioactive molecules, better known as “phytochemicals,” which aid in the protection of plants, and can have a huge impact on human health and metabolism [1]. A wide variety of medicinal properties are exhibited by different plants ranging from anti-inflammatory, antioxidant, antitumor and immunomodulatory effects [2], apart from improving cardiovascular ailments [3], treating kidney stones [4] and digestive diseases like inflammatory bowel disease [5], and many more. Another important advantage of using plants for their medicinal properties is that it either can be applied externally or can be consumed as a food or beverage [6–8].

Traditional medicine of countries varies depending upon the species of plants that grow in their vegetation and habitat. In ancient eras, traditional medicinal practices were followed to treat a variety of diseases such as headache, dizziness, cold, wounds, cough, and asthma. However, scientific advancements lead to the identification of bioactive compounds, which can induce these effects. The twentieth century witnessed the advancement of synthetic drugs and antibiotics over plant extracts in curing diseases because of its ease of using and quicker action. However, recently, many side effects of using these drugs surfaced which lead scientists to look back to the traditional way of using plants and their derivatives [1]. Until now, only a small percentage of the existing plant species have been scientifically explored for their bioactivities and possible benefits [9] which opens a wide arena in the field of research.

*Cleistocalyx nervosum* var. *paniala* (*C. nervosum*), an indigenous berry fruit widely grown in the northern parts of Thailand [10], belongs to the family Myrtaceae and is used in traditional medicine as it is known to possess various health benefits [11–15]. Additionally, it is a key ingredient in health drinks and functional foods, because of the characteristic sweet and sour taste along with the natural red color which contains anthocyanins, antioxidants, and phenolics [16]. *C. nervosum* is one of the richest sources of anthocyanins among various berry fruits [15, 17].

*C. nervosum* exhibit various medicinal properties and health benefits such as antioxidant and antiaging properties [16, 18], anticarcinogenic properties [11, 12], antiheavy metal toxicity [19], and antimicrobial activities [20, 21]. Our group has previously reported the antioxidant potential and neuroprotective effects of *C. nervosum* in HT22 cell lines [15]. However, there is no clear idea about the overall health benefits and the *in vivo* mechanism involved in attaining these effects.

The soil nematode *Caenorhabditis elegans* is widely used as a model to understand different parameters including aging, development, reproduction, stress resistance, immune enhancement, and neurological disorders [22–24]. *C. elegans* can be used to understand different neurotoxic disorders such as Alzheimer's [25], Parkinson's [26], and dementia [27]. Ease of handling and maintenance, short life cycle and life span, and availability of single-gene mutants make it one of the most preferred models [28]. *C. elegans* is the first eukaryotic organism to be completely sequenced [29]. Research in *C. elegans* using various nutraceuticals from plant sources has shed light on the involvement of several genes and pathways along with dietary interventions which can modulate lifespan and healthspan [30].

Many plants or plant derivatives such as green tea [31], tomatidine [32], *Streblus asper* [33], *Paullinia cupana* [34], Gengnianchun [35], and mulberry [36] were observed to extend lifespan and healthspan along with improving stress response and antioxidant mechanism in *C. elegans.* The present study tries to understand the effect of *C. nervosum* in extending lifespan and healthspan, incorporating neuroprotection along with improving stress resistance in *C. elegans*.

## 2. Materials and Methods

### 2.1. Chemicals, Reagents, and Equipment Used

All the chemicals and reagents used in the study were purchased from Sigma-Aldrich (St. Louis, MO, USA) and HiMedia Laboratories (Mumbai, India). *C. elegans* were exposed to UV-A for 4 h using a UV transilluminator lamp, SANKYO DENKI (F20T10BL).

### 2.2. Plant Collection, Extraction, and Detection of *In Vitro* Antioxidant Potential

Fruit pulp of *C. nervosum* was collected from ripe fruits from two different locations, Chiang Mai and Lampang, which will be designated as CMK-P and LMK-P, respectively, from now on. The pulp was freeze-dried, and then, 50 g of each powdered pulp was subjected to extraction with ethanol using the Soxhlet extraction method. The extraction was carried out for 2 days. Then, the extracts were concentrated at 50°C using a rotary evaporator, and the crude extract was further made as 100 mg/ml stock solution using dimethyl sulfoxide (DMSO) and stored at -20°C [15].

The *in vitro* antioxidant activity was monitored through a 2,2-diphenyl-1-picryl-hydrazyl-hydrate (DPPH) radical scavenging assay and oxygen radical absorbance capacity (ORAC) assay as described previously [15] by our group, and the results were represented as mg vitamin C (VC)/g dry sample and *μ*mol Trolox (TE)/g dry sample, respectively (*n* = 3).

### 2.3. *C. elegans* Strain Used and Culture Conditions

Wild-type strain N2 (Bristol), *daf-16* mutant CF1038, and A*β* transgenic strain CL2006 were purchased from the *Caenorhabditis* Genetics Center (University of Minnesota, USA) along with the bacterial food source *E. coli* OP50. All strains were maintained in nematode growth medium (NGM) at 15°C unless otherwise specified [37]. All the experiments were conducted in age-synchronized young adult worms. Each experiment was done in three independent trials [38].

### 2.4. Lifespan Assay

The lifespan assay was carried out, as explained previously [38]. The known number (~10) of age-synchronized nematodes (wild type and mutants) was placed in M9 buffer along with *E. coli* OP50 in a 24-well microtiter plate with different concentrations (1-100 *μ*g/ml for wild type and 10-40 *μ*g/ml for mutants) of *C. nervosum* fruit extracts dissolved in DMSO. The total number of live worms was counted every 24 h. 5-Fluoro-2′-deoxyuridine (FUDR) was added to prevent the production of progenies inside the experimental setup. Nematodes were considered dead when they do not respond even to a gentle tap or touch with the platinum loop. A parallel vehicle control of DMSO was also used, which was equivalent to the highest concentration of the solvent used. Worms treated only with *E. coli* OP50 were used as the control group. All the experiments were carried out in biological triplicates.

### 2.5. Pharyngeal Pumping Assay

The pharyngeal pumping assay was carried out, as explained previously [22]. The known number of young adult stage nematodes (~10) was transferred to NGM plates swabbed with different concentrations of *C. nervosum* fruit extracts. Pharyngeal pumping was observed once in every 24 h using a stereomicroscope (Motic SMZ-171) for 30 consecutive seconds. Pharyngeal pumping of worms in NGM plates swabbed with *E. coli* OP50 was considered as the control group.

### 2.6. Lipofuscin Imaging

Accumulation of autofluorescent proteins inside the nematode was done in wild-type nematodes treated with different concentrations of *C. nervosum* (20 and 30 *μ*g/ml) for 5 days. Worms treated only with *E. coli* OP50 were used as the control group. After incubation, the worms were washed thoroughly using M9 buffer and then transferred to a drop of sodium azide in a glass slide. Fluorescent imaging was done in 10 nematodes using a ZEISS LSM 700 confocal microscope using 10x magnification at the objective lens. The images were analyzed using ImageJ software, and the relative fluorescence was represented as arbitrary units (AU).

### 2.7. Measurement of Extracellular ROS Using DCF

Estimation of extracellular Reactive Oxygen Species (ROS) was done as previously described [38]. Briefly, two sets of wild-type nematodes were exposed to UV-A for 4 h. In the first set, the worms were treated with different concentrations of *C. nervosum* fruit extracts (20 and 30 *μ*g/ml) before exposure. In the second set, the worms were treated with different concentrations of *C. nervosum* fruit extracts (20 and 30 *μ*g/ml) after exposure. In both cases, the fruit extract treatment continued for 5 days and then were washed thoroughly with M9 buffer. After washing, the worms were incubated with 5 *μ*g/ml of DCFH-DA for 20 minutes, followed by another wash to remove the excess of DCFH-DA. Further, the worms were transferred to a drop of sodium azide in a glass slide. Fluorescent imaging was done in 10 nematodes using a ZEISS LSM 700 confocal microscope. The images were analyzed using ImageJ software, and the relative fluorescence was represented as arbitrary units (AU). Two controls were used, wherein one set was exposed to UV-A for 4 h and did not receive any extracts (positive control), and the other set had no exposure to UV-A and no extract treatment (negative control).

### 2.8. Total RNA Isolation and Real-Time PCR Analysis

The TRIzol kit (Invitrogen, Carlsbad, CA, USA) was used to isolate total RNA from wild-type nematodes treated with different concentrations of *C. nervosum* fruit extracts (20 and 30 *μ*g/ml). From the total RNA, 1000 ng was converted to cDNA using AccuPower RT Premix (Bioneer, Korea) with oligo dT primers following the manufacturer's protocol. Real-time PCR was carried out using SYBR Green, Green Star PCR Master Mix (Bioneer, Korea), in the Exicycler Real-Time Quantitative Thermal Block (Bioneer, Daedeok-gu, Korea) with the help of gene-specific primers. The expression data were normalized to the internal control actin and then represented as upregulated or downregulated by normalizing with the untreated control. The sequences of the primers are given in Table 1.

### 2.9. Statistical Analysis

Statistical analysis was carried out using GraphPad Prizm® for Mac version 6.0 h. All the results were represented as the mean ± standard deviation. *p* values lower than 0.05 were considered significant.

## 3. Results

### 3.1. *In Vitro* Antioxidant Potential of *C. nervosum* Extracts

The *in vitro* antioxidant potential was analyzed through DPPH and ORAC in *C. nervosum* extracts. The DPPH scavenging activity was observed to be 72.01 ± 3.32 mg VC/g dry sample and 104.19 ± 5.62 mg VC/g dry sample, respectively, for CMK-P and LMK-P extracts. Similarly, the ORAC levels were observed to be 140.17 ± 4.76 *μ*mol TE/g dry sample and 164.16 ± 5.45 *μ*mol TE/g dry sample, respectively, for CMK-P and LMK-P extracts (Table 2) indicating that LMK-P is with higher *in vitro* antioxidant activity when compared to CMK-P.

### 3.2. *C. nervosum* could Extend the Median and Maximum Lifespan of *C. elegans*

**Figure 1:**
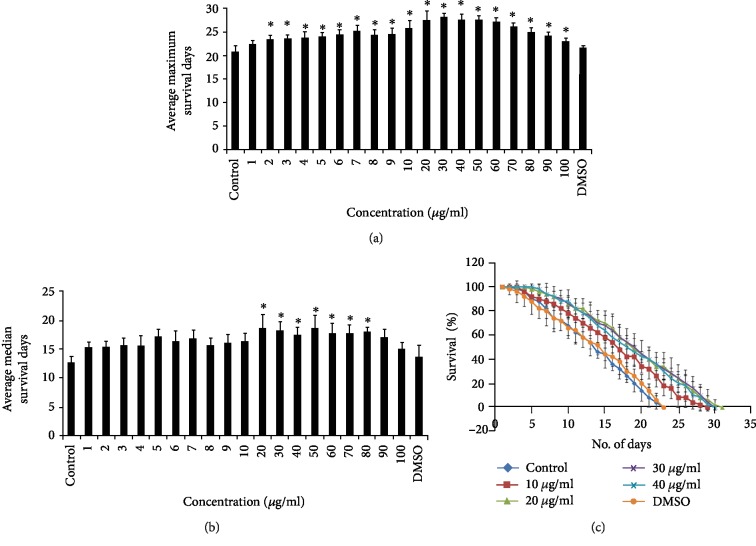
CMK-P extract can extend the mean and median lifespan of *C. elegans*. (a) Wild-type nematodes were treated with different concentrations of CMK-P extracts ranging from 1 to 100 *μ*g/ml which could significantly (*p* < 0.05) extend the maximum lifespan of the nematode. Nematodes used as the control which did not receive any extract treatment survived up to 22 days. DMSO was used as a vehicle control which also survived for 22 days. (b) Wild-type nematodes were treated with different concentrations of CMK-P extracts ranging from 1 to 100 *μ*g/ml which could significantly (*p* < 0.05) extend the median lifespan of the nematode. (c) Selective doses which showed maximum extension of lifespan were represented. CMK-P extracts at 10, 20, 30, and 40 *μ*g/ml could extend the lifespan of the nematode up to 28, 30, 29, and 29 days, respectively.

**Figure 2:**
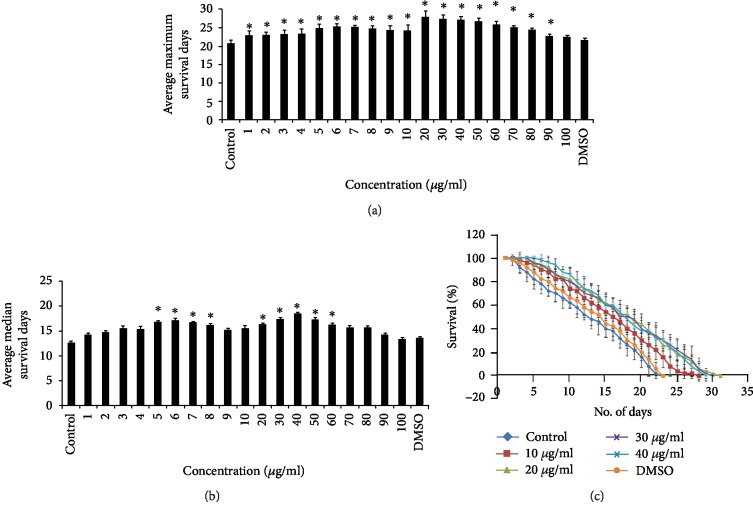
LMK-P extract can extend the mean and median lifespan of *C. elegans*. (a) Wild-type nematodes were treated with different concentrations of LMK-P extracts ranging from 1 to 100 *μ*g/ml which could significantly (*p* < 0.05) extend the maximum lifespan of the nematode. Nematodes used as the control which did not receive any extract treatment survived up to 22 days. DMSO was used as a vehicle control which also survived for 22 days. (b) Wild-type nematodes were treated with different concentrations of LMK-P extracts ranging from 1 to 100 *μ*g/ml which could significantly (*p* < 0.05) extend the median lifespan of the nematode. (c) Selective doses which showed maximum extension of lifespan were represented. LMK-P extracts at 10, 20, 30, and 40 *μ*g/ml could extend the lifespan of the nematode up to 27, 30, 29, and 28 days, respectively.

Both the *C. nervosum* extracts collected were able to extend the median and maximum lifespan of *C. elegans* in all the tested concentrations from 1 to 100 *μ*g/ml. Both CMK-P and LMK-P extracts exhibited an increase in median and maximum lifespan at all the tested concentrations (Figures 1 and 2). However, the higher doses of LMK-P, at 90 and 100 *μ*g/ml, could not increase the lifespan of the nematode; rather, it was similar to that of the control (Figure 2(a)). Doses ranging between 10 and 40 *μ*g/ml in both CMK-P and LMK-P showed maximum significance (*p* < 0.05) in increasing the maximum lifespan which was up to 28, 30, 29, and 29 and 27, 30, 29, and 28 days, respectively (Figures 1(c) and 2(c)). The worms used as the control, which was fed with laboratory food source *E. coli* OP50 and not treated with any of the extracts, survived up to 22 days (Figures 1 and 2). A parallel vehicle control was also used wherein the worms were treated with the highest dosage of solvent (DMSO) used, which also showed similar lifespan as of the control, indicating that no change was induced by the solvent (Figures 1 and 2).

### 3.3. *C. nervosum* Could Also Improve the Healthspan of *C. elegans*

**Figure 3:**
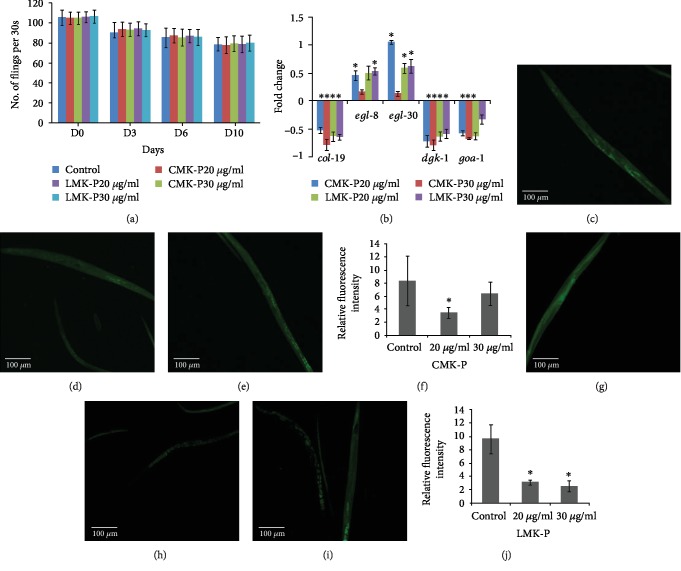
Both CMK-P and LMK-P could extend the healthspan of *C. elegans*. (a) Pharyngeal pumping assay of wild-type nematodes treated with CMK-P and LMK-P at 20 and 30 *μ*g/ml concentrations. Both the extracts did not show any significant change in the pharyngeal pumping rate when compared to the control. (b) qPCR analysis of *col-19*, *egl-8*, *egl-30*, *dgk-1*, and *goa-1* in wild-type nematodes treated with CMK-P and LMK-P. Both the extracts significantly (*p* < 0.05) downregulated the expression of *col-19*, *dgk-1*, and *goa-1* and upregulated the expression of *egl-8* and *egl-30*. (c) Representative image of wild-type nematode with no extract treatment (control) with the level of lipofuscin accumulation. (d) Representative image of wild-type nematode with 20 *μ*g/ml of CMK-P with the level of lipofuscin accumulation. (e) Representative image of wild-type nematode with 30 *μ*g/ml of CMK-P with the level of lipofuscin accumulation. (f) Relative fluorescence intensity comparison of nematodes treated with CMK-P extract at 20 and 30 *μ*g/ml showing significant (*p* < 0.05) reduction in fluorescence when compared to the control (*n* = 10). (g) Representative image of wild-type nematode with no extract treatment (control) with the level of lipofuscin accumulation. (h) Representative image of wild-type nematode with 20 *μ*g/ml of LMK-P with the level of lipofuscin accumulation. (e) Representative image of wild-type nematode with 30 *μ*g/ml of LMK-P with the level of lipofuscin accumulation. (f) Relative fluorescence intensity comparison of nematodes treated with LMK-P extract at 20 and 30 *μ*g/ml showing significant (*p* < 0.05) reduction in fluorescence when compared to the control (*n* = 10).

Pharyngeal pumping was analyzed in *C. elegans* treated with 20 and 30 *μ*g/ml of both CMK-P and LMK-P extracts. It was observed that both the extracts did not reduce the pharyngeal pumping of the nematodes and were showing a similar pumping rate when compared to the control worms fed with laboratory food source *E. coli* OP50 (Figure 3(a)).

The level of autofluorescent protein, lipofuscin, which is an indicator of aging, was monitored inside the nematodes treated with 20 and 30 *μ*g/ml of both CMK-P and LMK-P extracts. The LMK-P extract showed a significant (*p* < 0.05) reduction in the levels of lipofuscin in both the doses (Figures 3(g)–3(j)) whereas CMK-P showed significant (*p* < 0.05) reduction in 20 *μ*g/ml concentration when compared to the control (Figures 3(c)–3(f)), indicating that the extract could slow down or reduce the accumulation of this protein.

Further, qPCR analysis of candidate genes that mediate healthspan was monitored. It was observed that the expression of *egl-8* and *egl-30* was upregulated significantly (*p* < 0.05) and the expression of *col-19*, *dgk-1*, and *goa-1* was significantly (*p* < 0.05) downregulated in worms treated with 20 and 30 *μ*g/ml of both CMK-P and LMK-P extracts when compared to the control (Figure 3(b)).

### 3.4. *C. nervosum* Mediated Extension of Lifespan, and Healthspan Is Dependent and Independent of DAF-16 Pathway

**Figure 4:**
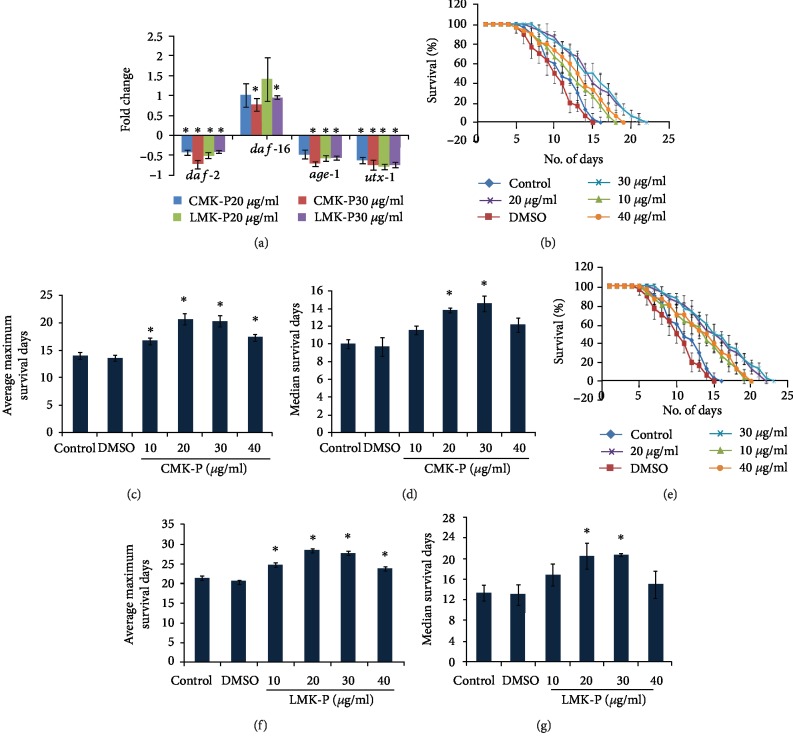
*C. nervosum* extracts are dependent and independent of DAF-16-mediated pathway. (a) qPCR analysis of candidate genes of DAF-16-mediated pathway. Wild-type nematodes treated with CMK-P and LMK-P extracts at 20 and 30 *μ*g/ml showed significant (*p* < 0.05) upregulation in the expression of *daf-16* at selective doses and corresponding significant (*p* < 0.05) downregulation of *daf-2*, *age-1*, and *utx-1*. (b) CMK-P at 10, 20, 30, and 40 *μ*g/ml could extend the maximum lifespan of *daf-16* mutants. (c) Graph showing significant increase in the average maximum survival days of *daf-16* mutant nematodes treated with 10, 20, 30, and 40 *μ*g/ml of CMK-P extracts. (d) Graph showing significant increase in the average median survival days of *daf-16* mutant nematodes treated with 10, 20, 30, and 40 *μ*g/ml of CMK-P extracts. (e) LMK-P at 10, 20, 30, and 40 *μ*g/ml could extend the maximum lifespan of *daf-16* mutants. (f) Graph showing significant increase in the average maximum survival days of *daf-16* mutant nematodes treated with 10, 20, 30, and 40 *μ*g/ml of LMK-P extracts. (g) Graph showing significant increase in the average median survival days of *daf-16* mutant nematodes treated with 10, 20, 30, and 40 *μ*g/ml of LMK-P extracts.

The qPCR analysis of major players of DAF-16 pathway was monitored in nematodes treated with 20 and 30 *μ*g/ml of both CMK-P and LMK-P extracts. It was observed that the expression of *daf-16* was upregulated significantly (*p* < 0.05) at 30 *μ*g/ml of both the extracts and that of *daf-2*, *age-1*, and *utx-1* was downregulated significantly (*p* < 0.05) when compared to the control, which indicated the role of DAF-16 pathway in *C. nervosum*-mediated extension of lifespan (Figure 4(a)).

In order to analyze the involvement of any other pathways in lifespan extension of *C. nervosum* extracts, mutants of DAF-16 were treated with 10–40 *μ*g/ml of both CMK-P and LMK-P extracts and the survival level was monitored. A significant (*p* < 0.05) increase in the median and maximum lifespan was observed at 20 and 30 *μ*g/ml concentration of both CMK-P (Figures 4(b)–4(d)) and LMK-P (Figures 4(e)–4(g)) extracts in the mutant worms. This suggests that some other mechanisms could also mediate the lifespan extension by *C. nervosum*.

### 3.5. *C. nervosum* can Activate the Antioxidant Potential inside *C. elegans*

**Figure 5:**
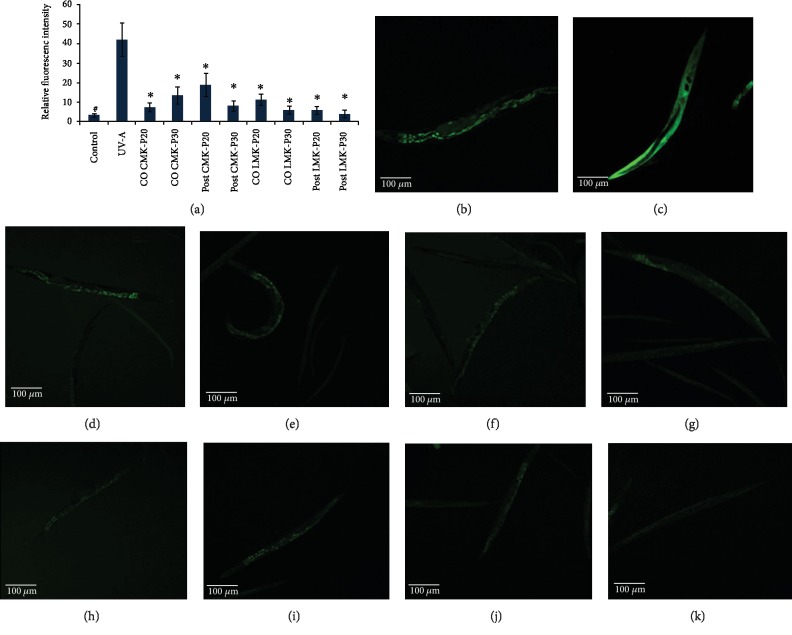
*C. nervosum* extracts could activate the antioxidant potential by reducing the level of ROS in *C. elegans*. (a) Relative fluorescence intensity comparison of nematodes exposed to UV-A for 4 h to induce stress along with co- and posttreatment with CMK-P and LMK-P extract at 20 and 30 *μ*g/ml showing significant (*p* < 0.05) reduction in fluorescence when compared to control worms exposed to UV-A without any extract treatment (*n* = 10). (b) Representative image of the negative control worm which was not exposed to UV-A and did not receive any extract treatment. (c) Representative image of the positive control worm which was exposed to UV-A for 4 h but did not receive any extract treatment. (d) Representative image of the worm which was treated with 20 *μ*g/ml of CMK-P along with UV-A exposure for 4 h (cotreatment). (e) Representative image of the worm which was treated with 30 *μ*g/ml of CMK-P along with UV-A exposure for 4 h (cotreatment). (f) Representative image of the worm which was treated with 20 *μ*g/ml of CMK-P after UV-A exposure for 4 h (posttreatment). (g) Representative image of the worm which was treated with 30 *μ*g/ml of CMK-P after UV-A exposure for 4 h (posttreatment). (h) Representative image of the worm which was treated with 20 *μ*g/ml of LMK-P along with UV-A exposure for 4 h (cotreatment). (i) Representative image of worm which was treated with 30 *μ*g/ml of LMK-P along with UV-A exposure for 4 h (cotreatment). (j) Representative image of the worm which was treated with 20 *μ*g/ml of LMK-P after UV-A exposure for 4 h (posttreatment). (k) Representative image of the worm which was treated with 30 *μ*g/ml of LMK-P after UV-A exposure for 4 h (posttreatment).

In order to analyze the antioxidant potential of *C. nervosum* extracts, *C. elegans* were induced with oxidative stress by exposing it to UV-A for 4 h [38]. The extracts were analyzed for the protective effects and repair effects by treating the extracts before and after induction of stress individually. There was a significant (*p* < 0.05) reduction of the oxidative stress level observed, which is directly proportional to the reduction in fluorescence, in both CMK-P and LMK-P extracts (Figure 5).

**Figure 6:**
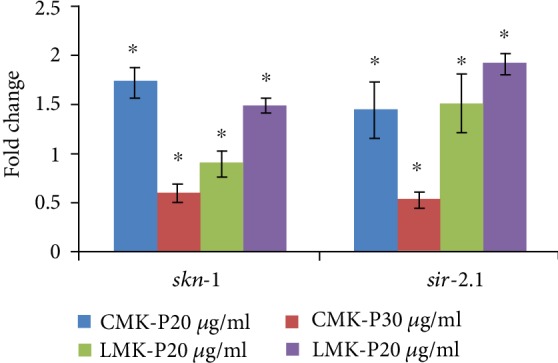
qPCR analysis of candidate genes *skn-1* and *sir-2.1* that mediate the antioxidant mechanism in *C. elegans*. Both *skn-1* and *sir-2.1* expressed significant (*p* < 0.05) upregulation in wild-type nematodes when treated with 20 and 30 *μ*g/ml of CMK-P and LMK-P extracts.

Further, qPCR analysis of *skn-1* and *sir-2.1*, candidate genes that mediate the antioxidant mechanism in *C. elegans*, was analyzed after treating with 20 and 30 *μ*g/ml of both CMK-P and LMK-P extracts. It was observed that the expression of both the genes was upregulated significantly (*p* < 0.05) indicating the activation of the antioxidant mechanism inside the nematode (Figure 6).

### 3.6. *C. nervosum* can Impart Neuroprotection in Transgenic *C. elegans*

**Figure 7:**
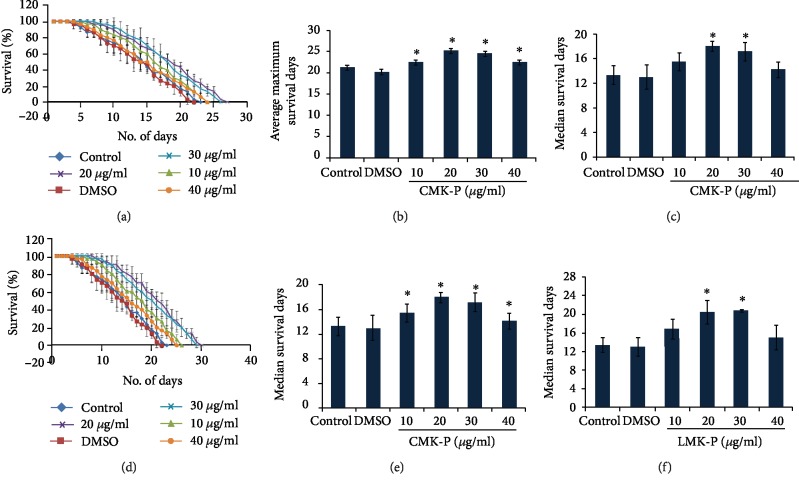
*C. nervosum* extracts could extend the survival of A*β* transgenic strain CL2006. (a) CMK-P at 10, 20, 30, and 40 *μ*g/ml could extend the maximum lifespan of A*β* transgenic strain. (b) Graph showing significant increase in the average maximum survival days of A*β* transgenic strain treated with 10, 20, 30, and 40 *μ*g/ml of CMK-P extracts. (c) Graph showing significant increase in the average median survival days of A*β* transgenic strain treated with 10, 20, 30, and 40 *μ*g/ml of CMK-P extracts. (d) LMK-P at 10, 20, 30, and 40 *μ*g/ml could extend the maximum lifespan of A*β* transgenic strain. (e) Graph showing significant increase in the average maximum survival days of A*β* transgenic strain treated with 10, 20, 30, and 40 *μ*g/ml of LMK-P extracts. (f) Graph showing significant increase in the average median survival days of A*β* transgenic strain treated with 10, 20, 30, and 40 *μ*g/ml of LMK-P extracts.

Finally, to analyze the neuroprotective effect of *C. nervosum* extracts, a transgenic strain of *C. elegans*, CL2006, which expresses A*β*_1-42_ constitutively was treated with 10–40 *μ*g/ml of both CMK-P and LMK-P extracts, and the survival level was monitored. It was observed that both CMK-P (Figures 7(a)–7(c)) and LMK-P (Figures 7(d)–7(f)) at 20 and 30 *μ*g/ml concentration could significantly (*p* < 0.05) extend the median and maximum lifespan of the nematodes suggesting its neuroprotective potential.

## 4. Discussion

Aging is the process of accumulation of damages to cells, tissues, and organs of an individual which is universal and unique, thereby reducing the overall health of the organism [39, 40]. Even after the advancements in the field of research, the complete mechanism of the aging process is yet unclear. Healthy aging depends on several broad factors such as physiological, biological, nutritional, behavioral, mental, and social factors [41]. From the available scientific knowledge, it is evident that aging can induce stress inside the system in the form of ROS or other stressors, reduce overall health, and induce age-associated neurological diseases [42–44]. In this regard, the focus is now on medicinal plants and its derivatives, which can exert antiaging potential by various diverse mechanisms including antioxidant, immune-enhancing, and neuroprotective potential with minimum side effects [44–47].

*C. nervosum* is one such plant that is reported to have immense antioxidant, antimutagenic, anticarcinogenic, and antiaging properties *in vitro* [11–15]. Our group has recently established this fruit to mediate neuroprotection in HT22 cells majorly based on its antioxidant potential, as it expressed free radical scavenging activity and antioxidant activity, which was evident from DPPH, ORAC, and FRAP assays. Further, cyanidin-3-glucoside was identified as the major anthocyanin, which could have many potential health benefits [15].

Aging is interconnected to lifespan even though both are not equivalent [39]. Lifespan alone can determine the overall survival rate of the organism, although it cannot clearly define the rate of aging [48]. In this regard, it is important to analyze the lifespan along with healthspan to determine the antiaging properties [39, 48]. In the present study, *C. nervosum* was able to extend the median and maximum lifespan of *C. elegans* in all the concentrations tested from 1 to 100 *μ*g/ml (Figures 1 and 2), except the higher concentrations of LMK-P at 90 and 100 *μ*g/ml, where there was no increase in lifespan and these doses showed similar effects when compared to the control (Figure 2). This suggests that the extracts of *C. nervosum* are not toxic and can improve the lifespan in *C. elegans*. Interestingly, selective doses of both the extracts were observed to significantly (*p* < 0.05) increase the lifespan (Figures 1 and 2). Many plant extracts and bioactive compounds involved such as *Paullinia cupana* [34], mulberry anthocyanins [36], *Momordica charantia* [49], *Gastrodia elata* [50], *Baccharis trimera* [51], and *Polygonum multiflorum* [52] were known to extend lifespan in *C. elegans* and various other models and express antiaging potential.

Interestingly, in *C. elegans*, a dietary restriction or calorie restriction process can be activated, which can also extend lifespan, which is interconnected to many other pathways [39, 53, 54]. In order to confirm that the lifespan extension observed was not mediated by dietary restriction, the pharyngeal pumping assay was carried out in *C. elegans* treated with *C. nervosum* extracts. It was observed that there was no difference in the pharyngeal pumping rate in worms fed with *C. nervosum* extracts when compared to the control (Figure 3(a)). This suggests that there was no dietary restriction mechanism involved in *C. elegans* when treated with *C. nervosum* extracts.

Healthspan is another key parameter that has to be monitored to analyze the antiaging potential [39, 48]. In the present study, the level of lipofuscin in the nematode treated with *C. nervosum* extracts was monitored. Lipofuscin is also known as “age pigment” which is a conserved autofluorescent protein which accumulates over the aging of an organism as it consists of nondegradable, highly oxidized materials [55]. It was observed that the LMK-P extract could significantly (*p* < 0.05) reduce the levels of lipofuscin in both the tested doses (Figures 3(g)–3(j)) whereas CMK-P could significantly (*p* < 0.05) reduce in 20 *μ*g/ml concentration when compared to the control (Figures 3(c)–3(f)). Previous reports suggest that the plant extracts which have antiaging potential can reduce the accumulation of lipofuscin in the nematode [52, 56–58].

Further, to confirm the activation of healthspan, qPCR analysis of candidate genes that regulate healthspan was monitored. In *C. elegans*, *col-19* is considered as an adult-specific marker [59, 60] as its expression starts from the late larval stages and increases as it reaches adulthood [22, 61]. In the present study, the expression of *col-19* was observed to be downregulated significantly (*p* < 0.05) in the nematodes treated with selective doses of both *C. nervosum* extracts when compared to the control (Figure 3(b)) indicating the antiaging potential of *C. nervosum*.

The diacylglycerol (DAG) pathway which constitutes the orthologs of Go ligands (*egl-8*, *egl-30*, *goa-1*, and *dgk-1*) is essential for healthspan [22, 62] including pharyngeal pumping, locomotion, and egg-laying wherein *egl-8* and *egl-30* are regulating positively [63] and *dgk-1* and *goa-1* are regulating negatively [64, 65]. Additionally, the serotonin biosynthesis in *C. elegans* is also mediated by *egl-30* and *goa-1* [66]. In the present study, the qPCR expression of *egl-8* and *egl-30* was observed to be upregulated significantly (*p* < 0.05) at all doses except 30 *μ*g/ml of CMK-P extract whereas the expression of *dgk-1* and *goa-1* was observed to be significantly (*p* < 0.05) downregulated (Figure 3(b)). This confirms that *C. nervosum* extracts can extend the healthspan of the nematode.

Further, to understand the pathway that regulates the extension of lifespan and healthspan mediated by *C. nervosum*, the role of DAF-16-mediated pathway was investigated. The insulin/IGF-1 signaling (IIS) pathway, commonly known as DAF-16-mediated pathway, is an evolutionarily conserved pathway which is one of the first major pathways to be identified to regulate the aging process in *C. elegans*. The pathway majorly comprises of *daf-2*, orthologous to IIS receptor, *age-1*, orthologous to PI-3-kinase, and *daf-16*, orthologous to the FOXO (Forkhead Box O) transcription factor. Mutations in *daf-2* or *age-1* can increase the lifespan, whereas mutations in *daf-16* can reduce the lifespan. The pathway is interconnected to many other pathways or transcription regulators such as SKN-1, HSF-1, JNK-1, and mTOR [39, 48, 67]. UTX-1 constitutes to a conserved family of histone demethylases specific for lysine 27 of histone H3 (H3K27me3). RNAi of *utx-1* extended the lifespan of the nematode, which was dependent on DAF-16 [68, 69]. It is also crucial for embryonic and postembryonic development of *C. elegans* [70].

The qPCR expression of *daf-2*, *daf-16*, *age-1*, and *utx-1* was analyzed in *C. elegans* treated with *C. nervosum* extracts. It was observed that the expression of *daf-16* was upregulated significantly (*p* < 0.05) at 30 *μ*g/ml of both the extracts whereas the expression of *daf-2*, *age-1*, and *utx-1* was downregulated significantly (*p* < 0.05) in all the concentrations tested of both *C. nervosum* extracts (Figure 4(a)). This suggests that the lifespan extension mediated by *C. nervosum* extracts could be dependent of the DAF-16-mediated pathway. Since the DAF-16-mediated pathway is interconnected to many different pathways and mechanisms [39, 48, 67], the effect of *C. nervosum* extracts on the lifespan of *daf-16* mutants was analyzed. Interestingly, it was observed that there was a significant (*p* < 0.05) increase in the median and maximum lifespan of the mutants when treated with both CMK-P (Figures 4(b)–4(d)) and LMK-P (Figures 4(e)–4(g)). This suggests that the lifespan extension induced by *C. nervosum* extracts could be dependent and independent of the DAF-16-mediated pathway.

A previous study suggests that 14-3-3 protein can activate stress resistance in *C. elegans* in a DAF-16-dependent and DAF-16-independent manner [71]. The other transcription factors that regulate longevity in *C. elegans* in both DAF-16-dependent and DAF-16-independent-mediated pathways are mTOR and SKN-1 [39, 67]. The mTOR may regulate longevity either by mediating DAF-16 or via SKN-1 [72]. This suggests the involvement of the transcription factor SKN-1, which is responsible for the antioxidant mechanism in the nematodes treated with *C. nervosum*.

Our group has previously observed the antioxidant properties of *C. nervosum* extract *in vitro* [15]. In this regard, in the present study, the antioxidant activity of *C. nervosum* extracts was validated *in vivo* by monitoring the ability of the extract to reduce the level of oxidative stress formed inside *C. elegans*. Wild-type nematodes were exposed to UV-A for 4 h, which can induce oxidative stress in *C. elegans* [38]. The protective effect and the repair effect of *C. nervosum* extracts were analyzed by treating the extracts to the nematodes during the course and after UV-A exposure, individually. Interestingly, it was observed that in both the cases, there was a significant (*p* < 0.05) reduction in the level of oxidative stress in both CMK-P and LMK-P extract-treated nematodes when compared to those which were exposed to UV-A without any extract treatment which was evident from the reduction in fluorescence that is proportional to the level of oxidative stress inside the nematode (Figure 5). Recent studies report that several plant extracts can activate antioxidant metabolism mediated by *skn-1* to activate antiaging and stress resistance mechanisms [34, 36, 51, 73].

The qPCR expression of two candidate genes which are responsible for antioxidant activity, *skn-1* and *sir-2.1*, was analyzed in wild-type nematodes treated with *C. nervosum* extracts to further confirm the effects. It was observed that both the genes were upregulated significantly (*p* < 0.05) (Figure 6) indicating the activation of the antioxidant mechanism. Orthologous to mammalian sirtuins, *sir-2.1* in *C. elegans* is known to activate the antioxidant mechanism during oxidative stress. Different extracts or compounds such as green tea [31], black tea [74], resveratrol [75], emodin [76], *Polygonum multiflorum* [58], and *Paullinia cupana* [77] were found to induce antioxidant effects in *C. elegans* via *sir-2.1*.

The use of antioxidants has recently emerged as a potential treatment option for neurological disorders, since oxidative stress was identified as one of the causes or relative after-effect of neurological disorders [78]. Many plant extracts such as *Paullinia cupana* [34] and *Baccharis trimera* [51] were observed to protect the *C. elegans* model for Alzheimer's disease from A*β*-mediated toxicity along with antioxidant properties. Our group has also reported the neuroprotective effect of *C. nervosum* extract from glutamate-induced toxicity in HT22 cell lines [15]. In this regard, in the present study, transgenic strains of *C. elegans*, which can be used as a model for Alzheimer's disease, were treated with *C. nervosum* extracts and observed for their survival. Interestingly, it was observed that both the extracts at 20 and 30 *μ*g/ml concentrations could significantly (*p* < 0.05) extend the median and maximum lifespan of the worms and keep them in an active state when compared to the control (Figure 7) indicating that the extract could elicit neuroprotective effect in nematodes. Various plant metabolites have been reported to elicit positive effects against neurological diseases by reducing plaque formation, improving memory and learning, reducing A*β* load from the blood-brain barrier, and improving cognitive functions [79].

The activation of SKN-1 by *C. nervosum* could have played a major role in extending lifespan and healthspan along with improving stress resistance and neuroprotection [80]. Recent reports suggest that plant extracts and its derivatives such as *Paullinia cupana* [34], rose essential oils [81], and *Cratoxylum formosum* [82] were able to elicit neuroprotection which is mediated via SKN-1-regulated antioxidant response. However, further in-depth molecular studies are required to validate that these effects showed by *C. nervosum* extracts were dependent on SKN-1.

Additionally, it is also important to note that the two extracts used in the present study were collected from two different provinces in Thailand, Chiang Mai, and Lampang. It was observed from the results that the fruit from Lampang Province had greater *in vitro* antioxidant potential when compared to that from Chiang Mai (Table 2). In the *in vivo* experiments using *C. elegans*, LMK-P at the tested concentrations of 20 and 30 *μ*g/ml was able to significantly reduce the level of lipofuscin accumulation whereas CMK-P was able to express significant reduction only at 20 *μ*g/ml concentrations (Figure 3). *Solanum aethiopicum* collected from two different locations in Nigeria expressed variations in phenolic profile, polyphenol contents, antioxidant activities, and enzyme inhibitory properties [83]. The *in vitro* antioxidant activity analyzed in *Withania somnifera* collected from two different locations in India [84] was also not similar to each other. The differences were mainly observed in the antioxidant activity which could be attributed to the differences in the geographical location and the habitat. However, both the extracts exhibited similar effects in significantly extending the lifespan in wild-type and mutant worms along with mediating the expression of candidate genes that mediate antiaging and stress resistance.

## 5. Conclusion

Altogether, *C. nervosum* extracts were found to be nontoxic to *C. elegans*, and optimum doses were observed to extend lifespan and healthspan significantly. This effect was dependent and independent of DAF-16 which was evident through qPCR and mutant-based analysis. The extract was also able to activate the antioxidant mechanism as it reduced the level of ROS and activated the expression of *skn-1* and *sir-2.1* which was confirmed through qPCR analysis. The activation of the antioxidant mechanism could have aided in the neuroprotective effect which allowed lifespan extension in the *C. elegans* model for Alzheimer's disease. Overall, *C. nervosum* extracts, which are used in the food industry [85], can be promoted as a major food additive with antiaging, antioxidant, and neuroprotective efficacies.

## Figures and Tables

**Table 1:** List of primers used.

Gene name	Forward primer	Reverse primer
*daf-2*	TCGAGCTCTTCCTACGGTGT	CATCTTGTCCACCACGTGTC
*daf-16*	TGGTGGAATTCAATCGTGAA	ATGAATATGCTGCCCTCCAG
*age-1*	ATAGAGCTCCACGGCACTTT	ATAGAGCTCCACGGCACTTT
*utx-1*	GCAGAACACCAGCTCATCAG	ATCAACGCCATTCTTCTCGC
*col-19*	CACACAAATGCTCCACCAAC	CTGGATTTCCCTTCTGTCCA
*egl-8*	CGTATCGTTGCGCTTCTCA	AGTAGTGACACAGCGGTTG
*egl-30*	TCAGAAAGGCGGAAGTGGAT	GGTTCTCGTTGTCACACTCG
*dgk-1*	GTTGGGGAAGTGGTGCAAAT	GCGAGCTTGGATTGGATGAG
*goa-1*	TGTTCGATGTGGGAGGTCAA	TCGTGCATTCGGTTTGTTGT
*skn-1*	ATCCATTCGGTAGAGGACCA	GGCGCTACTGTCGATTTCTC
*sir-2.1*	CGGGGAAGTGCAAGAAATAA	GAGTGGCACCATCATCAAGA
*act-2*	ATCGTCCTCGACTCTGGAGATG	TCACGTCCAGCCAAGTCAAG

**Table 2:** *In vitro* antioxidant potential of *C. nervosum* extracts.

Extract used	DPPH (mg VC/g dry sample)	ORAC (*μ*mol TE/g dry sample)
CMK-P	72.01 ± 3.32	140.17 ± 4.76
LMK-P	104.19 ± 5.62	164.16 ± 5.45

## Data Availability

All the data used to support the findings of this study are available from the corresponding author upon request.
